# Precise positioning of cancerous cells on PDMS substrates with gradients of elasticity

**DOI:** 10.1007/s10544-016-0110-y

**Published:** 2016-09-13

**Authors:** J. Raczkowska, S. Prauzner-Bechcicki

**Affiliations:** 1 The Marian Smoluchowski Institute of Physics, Jagiellonian University, Łojasiewicza 11, 30-428 Kraków, Poland; 2 Institute of Nuclear Physics Polish Academy of Sciences, Radzikowskiego 152, 31-342 Kraków, Poland

**Keywords:** Precise cell positioning, PDMS, Patterns of elasticity, Elasticity gradient

## Abstract

In this work the novel method to create PDMS substrates with continuous and discrete elasticity gradients of different shapes and dimensions over the large areas was introduced. Elastic properties of the sample were traced using force spectroscopy (FS) and quantitative imaging (QI) mode of atomic force microscopy (AFM). Then, fluorescence microscopy was applied to investigate the effect of elastic properties on proliferation of bladder cancer cells (HCV29). Obtained results show that cancerous cells proliferate significantly more effective on soft PDMS, whereas the stiff one is almost cell-repellant. This strong impact of substrate elasticity on cellular behavior is driving force enabling precise positioning of cells.

## Introduction

Recent research, also theoretical (Lange and Fabry 2013; Schwarz and Safran 2002), show that substrate elasticity affect strongly lot of cellular processes, such as spread area, cytoskeletal structure, proliferation, differentiation (Eroshenko et al. 2013; Evans et al. 2009; Guvendiren and Burdick 2012; Palchesko et al. 2012; Prauzner-Bechcicki et al. 2015; Sun et al. 2012;), or cell stiffness (Ghosh et al. 2007; Paszek et al. 2005; Schwarz and Bischofs 2005; Solon et al. 2007; Johnson and Harley 2011).

Moreover, elastic properties are highly relevant to numerous biological phenomena - matrix stiffening accompanies aging, cardiovascular disease and wound healing (Assoian and Klein 2008; Bhatia 2012; Kumar and Weaver 2009; Ng and Brugge 2009; Zhu et al. 2012). Also tumor growth and metastasis are related to the ability of cancerous cells to sense and adapt to mechanical forces of the microenviroment (Kumar and Weaver 2009) - tumor tissues have altered elastic properties, as compared to proper tissues (Assoian and Klein 2008; Kumar and Weaver 2009; Lekka et al. 1999; Lekka 2012; Nelson and Bissell 2006; Ng and Brugge 2009; Schedin and Keely 2010; Yu et al. 2010; Zhu et al. 2012). Increased elasticity of extracellular matrix is even believed to be the driving force of oncogene activity (Paszek et al. 2005; Saez et al. 2007; Zaman et al. 2006).

The overwhelming majority of the studies focuses on the influence of substrate with uniform elasticity on cellular behavior. However, in living organisms, cells response also to gradients of stimuli (Gray et al. 2002; Wu et al. 2012). They sense local elasticity, varying even few orders of magnitude, especially at the interfaces – e.g. between hard bone and soft cartilage (Engler et al. 2006; Kandow et al. 2007; Seidi et al. 2011; Sunyer et al. 2012; Tse and Engler 2011). These elasticity variations have profound effect on cellular behavior (Engler et al. 2006; Kandow et al. 2007; Seidi et al. 2011; Sunyer et al. 2012; Tse and Engler 2011) and have been suggested to guide the migration of cancer cells towards sites of intravasation (Kumar and Weaver 2009). Therefore, to study the influence of mechanic properties of the environment on the cellular behavior, it is essential to introduce substrates with elasticity gradient, i.e. with mechanical properties changing continuously or discrete over a certain distance across the substrate (Genzer 2012; Wu et al. 2012).

Moreover, substrates with discrete elasticity patterns may be used for spatial ordering of cells. Precise positioning of cells is a challenging issue for *in vitro* research and mimicking *in vivo* cellular microenviroment (Moustafa et al. 2014; Walker et al. 2004; Wang et al. 2009), essential for basic studies on intercellular interactions as well as for potential applications in cell-based biosensors and microarrays (Gonzalez-Macia et al. 2010; Hwang et al. 2009). Spatial control of cellular growth enables better understanding of how cells communicate and order in complex tissues, which is a key question for regenerative medicine and transplantology (Moustafa et al. 2014; Schwarz and Bischofs 2005).

In this paper we propose fast, simple and not expensive method to prepare polydimetyhylosiloxane (PDMS) substrates with both, continuous and discrete gradients of elasticity over large areas, simply by UV irradiation through printed mask. This technique enables fabrication of PDMS substrates with elasticity patterns in a wide range of spatial dimensions and shapes, limited only by printer resolution, i.e. > 50 μm. Mechanical properties of substrates, measured using force spectroscopy (FS) mode of atomic force microscopy (AFM), are modulated exclusively, leaving other physico-chemical PDMS properties unchanged (Prauzner-Bechcicki et al. 2015; Raczkowska et al. 2016).

These substrates are used to locate bladder cancer cells (HCV29) precisely. The positioning of cells, investigated using fluorescence microscopy is driven by preferential adhesion and proliferation of cells to softer areas and cell-repellant character of the stiffer regions. This approach enables an easy way to control cells spatially and to localize them in desired patterns, not only giving another tool to study basic cellular properties, but also promising method which could be used in potential application in cell-based biosensors or microarrays.

## Experimental

### PDMS substrate preparation

The PDMS mixture was prepared using commercially available Sylgard 184 (Dow Corning) with the base: curing agent mass ratio of 10:1. Next, it was admixed with benzophenone (Sigma-Aldrich) at the mass ratio of 1:100 benzophenone: PDMS dissolved in xylene (200 mg/ml, POCH Gliwice), and degassed.

The PDMS substrates were prepared on the 25 mm round coverslip glass using a spin-coating technique, resulting in PDMS films with thickness of ~60 μm. The spinning speed was 500 rpm. Then, a fraction of substrates was exposed to UV light (400 W mercury lamp) for times ranging from 0 to 5 h. The irradiated and non-irradiated substrates were baked for 15 min at 150 °C.

### PDMS with gradient elasticity

**Fig. 1:**
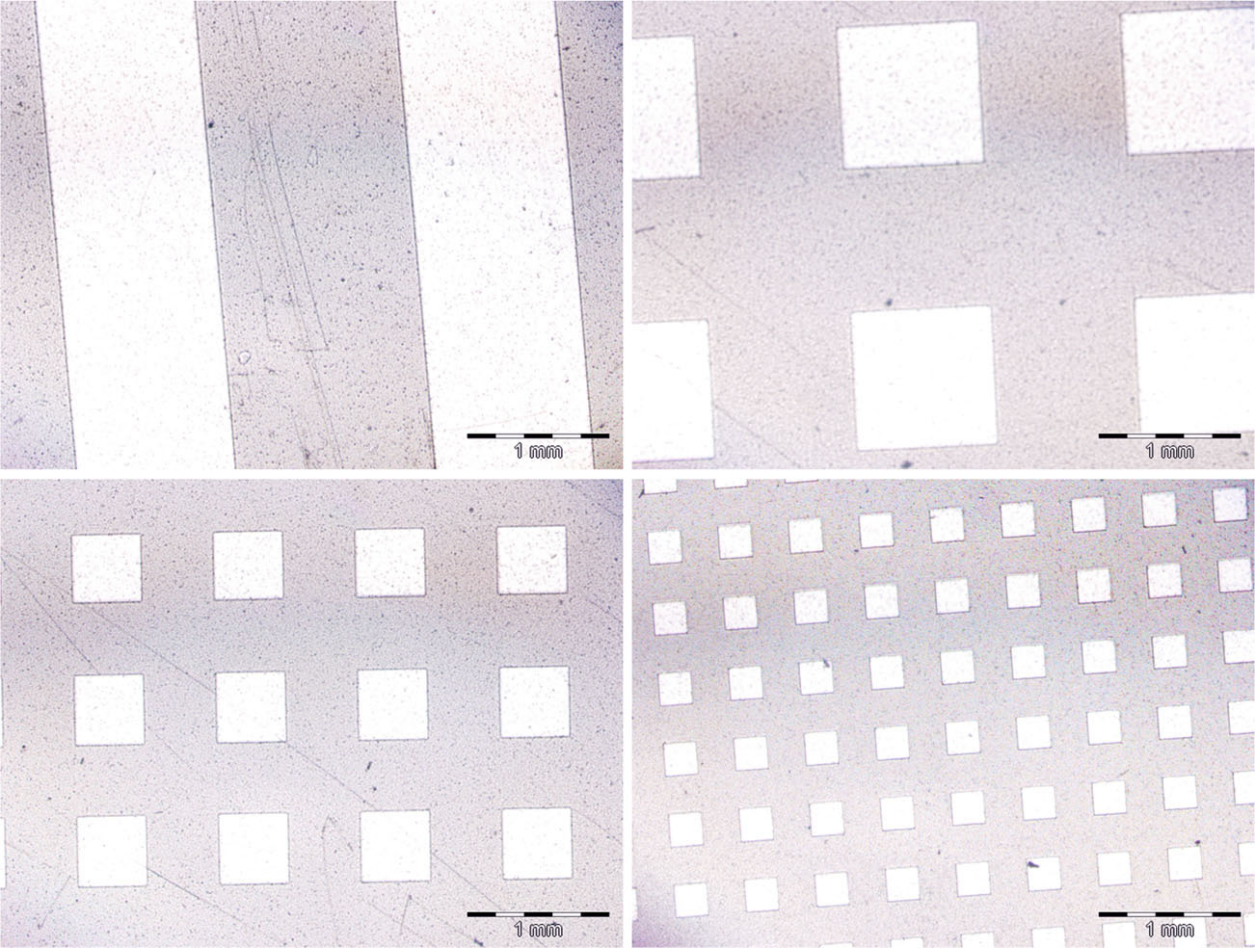
Exemplary masks for gradient elasticity patterns, printed on the foil

To prepare substrates with gradient elasticity, PDMS was first spun-cast onto 75 × 26 mm microscope slides. Then, the substrates were exposed to UV light (400 W mercury lamp) through the mask with desired pattern, printed simply on the foil (Fig. 1). After 5 h of UV exposure, substrates were baked for 15 min at 150 °C.

Alternatively, to obtain continuous elasticity gradient, substrates were completely covered with the shutter, which subsequently moved with constant speed (v = 0.0036 mm/s), exposing gradually PDMS substrate to UV light. Then, the substrates were baked for 15 min at 150 °C.

### Force spectroscopy

Commercially available AFM system AFM-5500 from Keysight (USA) was used to perform force spectroscopy experiments. The PDMS substrates were mounted in the AFM liquid cell. Then the 500 μl of the distilled water was gently put on a top of the measured surface. Afterwards the AFM tip was immersed in the liquid and brought close to the surface. A grid of 8 × 8 force-distance curves was collected in three different places for each sample.

From the recorded approach part of the force curves, i.e. dependencies of a load force and a relative displacement, a slope was determined from a linear regression. The slope denotes the sample stiffness expressed in N/m.

### Imaging of elastic properties

Imaging of local stiffness properties of a substrate was performed using Nanowizard AFM (JPK, Germany). Imaging was done using quantitative imaging (QI) mode, which gives the possibility to collect a force-distance curve at each pixel of scanned area. As a results map of mechanical properties is collected for given substrate. Representative AFM micrographs 10 × 10 μm^2^ (64 × 64 pixels) size were recorded. All collected force distance curves were analyzed in order to get information about the distribution of substrate stiffness at microscale area (100 μm^2^). QI measurements were performed in liquid environment.

### Cell culture

HCV-29 cell line was cultured in RPMI-1640 medium (Sigma) supplemented with 10 % fetal calf serum (FCS, Gibco) in culture asks, in a CO_2_ incubator provided 5 % air/95 % CO_2_ atmopsphere. The PDMS substrates on glass coverslips were put into Petri. Then, they were sterilized for one hour under UVC light (germicidal lamp, λ = 254 nm) under a laminar flow chamber (Nu425 from NuAire).

After sterilization, solution with cells (80,000 cells per ml) was introduced. Next, the Petri dish was moved into the CO_2_ incubator for 1, 3, or 6 days. The experiments were repeated at least 2 times for each time-point.

### Cell staining

To stain cells, the following procedure was applied. First, cells that were cultured on PDMS substrates with different elasticity were pre-fixed by adding to the culture medium 1 ml of the solution of 3:7 % of paraformaldehyde (Fluka) for 2 min at 37 ˚C. Then, cells were washed with phosphate buffered saline (PBS, Sigma Aldrich) 3 times for 2 min. Afterwards, to fix cells firmly, the sample was immersed in the solution of 3:7 % of paraformaldehyde (Fluka) for 15 min at room temperature. After removing the fixative, the cells were washed again with the PBS buffer (3 × 2 minutes). Next, the cells were incubated with the 0:2 mg/mL solution of Triton X-100 for 5 min at 4 ˚C. Afterwards, the cells were washed with PBS in the same way as previously described (3 × 2 min). For actin cytoskeleton staining, the Alexa-Fluor 488 (absorption maximum 488 nm - blue light, and emission 518 nm - green) conjugated with phalloidin (0:033 μM in PBS, Molecular Probes) was added for 30 min in dark and washed in PBS again (3 × 2 min). The cell nuclei have been labeled using the Hoechst dye (the absorption at 355 nm - ultraviolet, and the emission at 465 nm - blue). The Hoeschst solution (1 mg/mL in PBS, Sigma Aldrich) was added for 15 min in dark, followed by washing in PBS buffer (3 × 2 min).

### Image recording

Fluorescent images were recorded using optical microscope Olympus IX51 equipped with a 100 W Mercury light source (Olympus U-LH100HG), U-MWIG2 filter (λexit =530 - 550 nm, λemit =590 nm) and U-MNB2 one (λexit =470 - 490 nm, λemit =520 nm). The first filter was used to record image of actin filaments while the later one to detect fluorescently-labeled cell nuclei. Fluorescent images were recorded using the XC30 digital camera (Olympus). The maximum resolution of captured image by this camera has a 2080 × 1544 px. All images were recorded using CellSense Dimensions (Olympus) software with the objective 20× (Universal Plan Fluorite, magnification of 200).

## Results and discussion

### Cells on uniform PDMS substrates with different elasticity

Mechanical properties of PDMS substrates, tuned by time of UV irradiation, were measured using AFM-based force spectroscopy, providing Young modulus equal to 0.24 and 1.67 MPa for soft (UV irradiated for 5 h) and stiff (not irradiated) PDMS, respectively. Other physicochemical properties of the substrate, i.e. chemical composition, wettability, surface energy and topography were not affected by the tuning of elasticity procedure (Raczkowska et al. 2016).

**Fig. 2:**
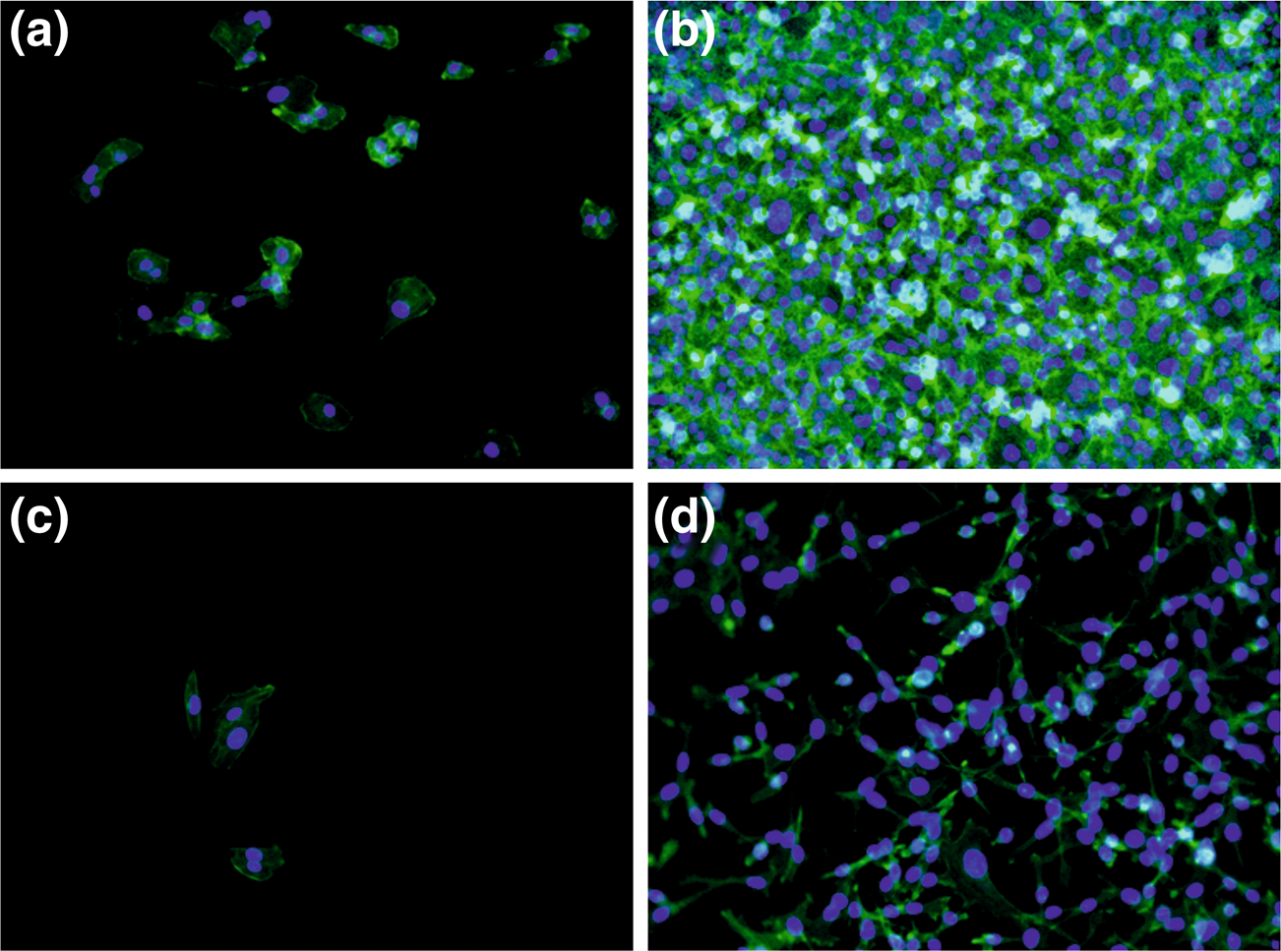
Fluorescence micrographs of HCV29 cells on soft (**a,** b) and stiff (**c, d**) PDMS after 24 (**a, c**) and 144 h (**b, d**) of incubation

To investigate potential effect of elasticity on cellular behavior, non-malignant HCV29 cells were cultured onto sof and stiff PDMS substrates. The representative fluorescence micrographs, recorded after 24 and 144 h of culture are presented in Fig. 2.

**Fig. 3:**
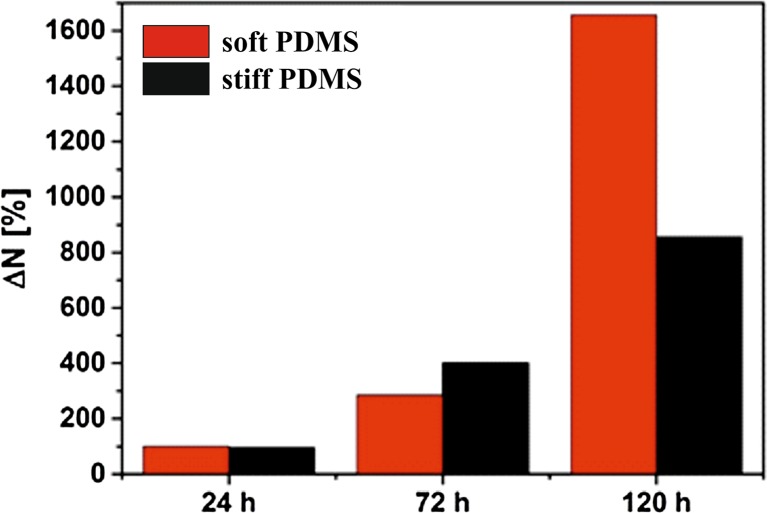
The relative change ΔN in number of HCV29 cells on softer and stiffer PDMS (all data are normalized do the number of cells on softer PDMS after 24 h in culture)

**Fig. 4:**
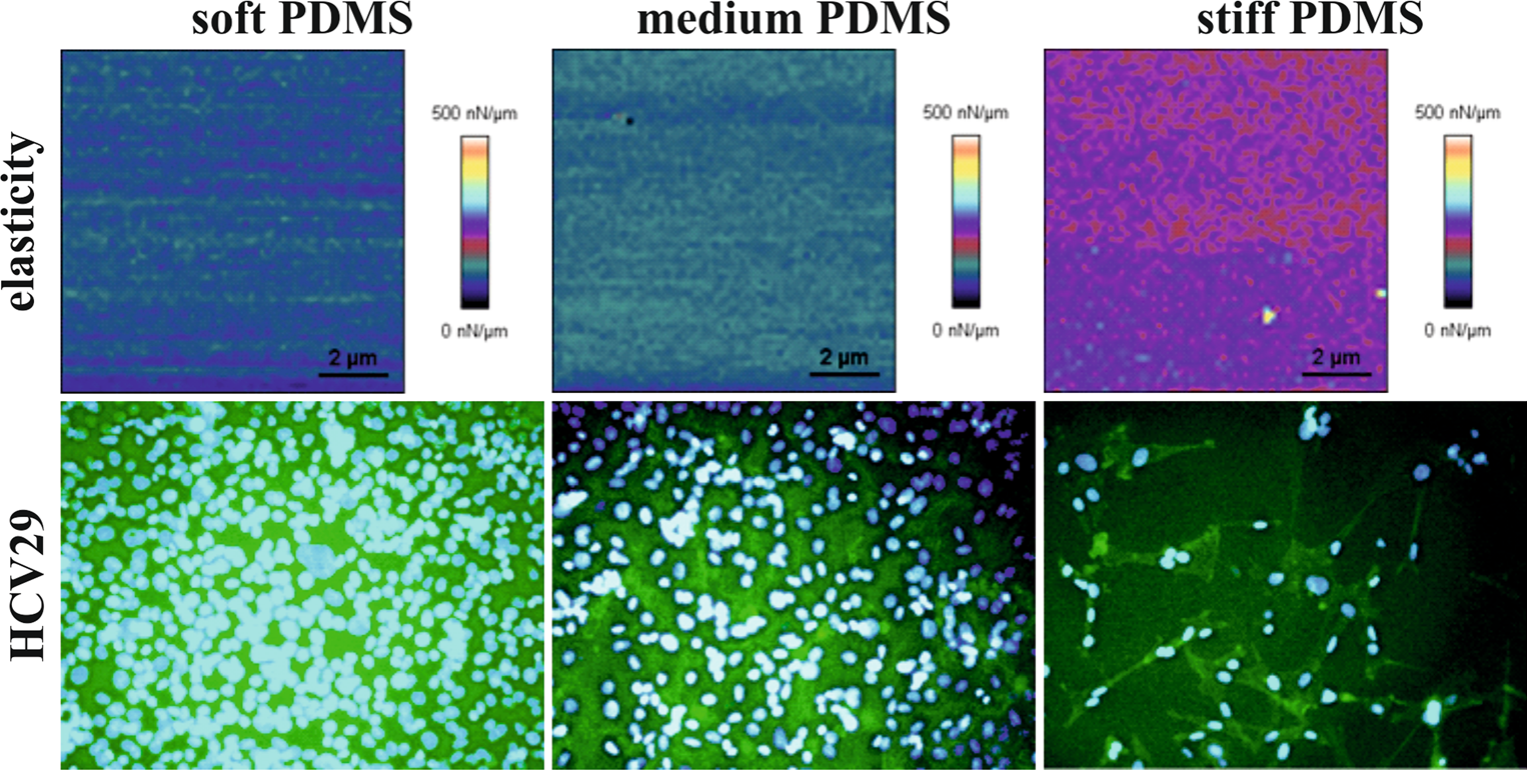
Elasticity maps (*upper row*) and fluorescence micrographs of proliferation of non-malignant HCV29 (*bottom row*) cells on substrates with elasticity gradient

**Fig. 5:**
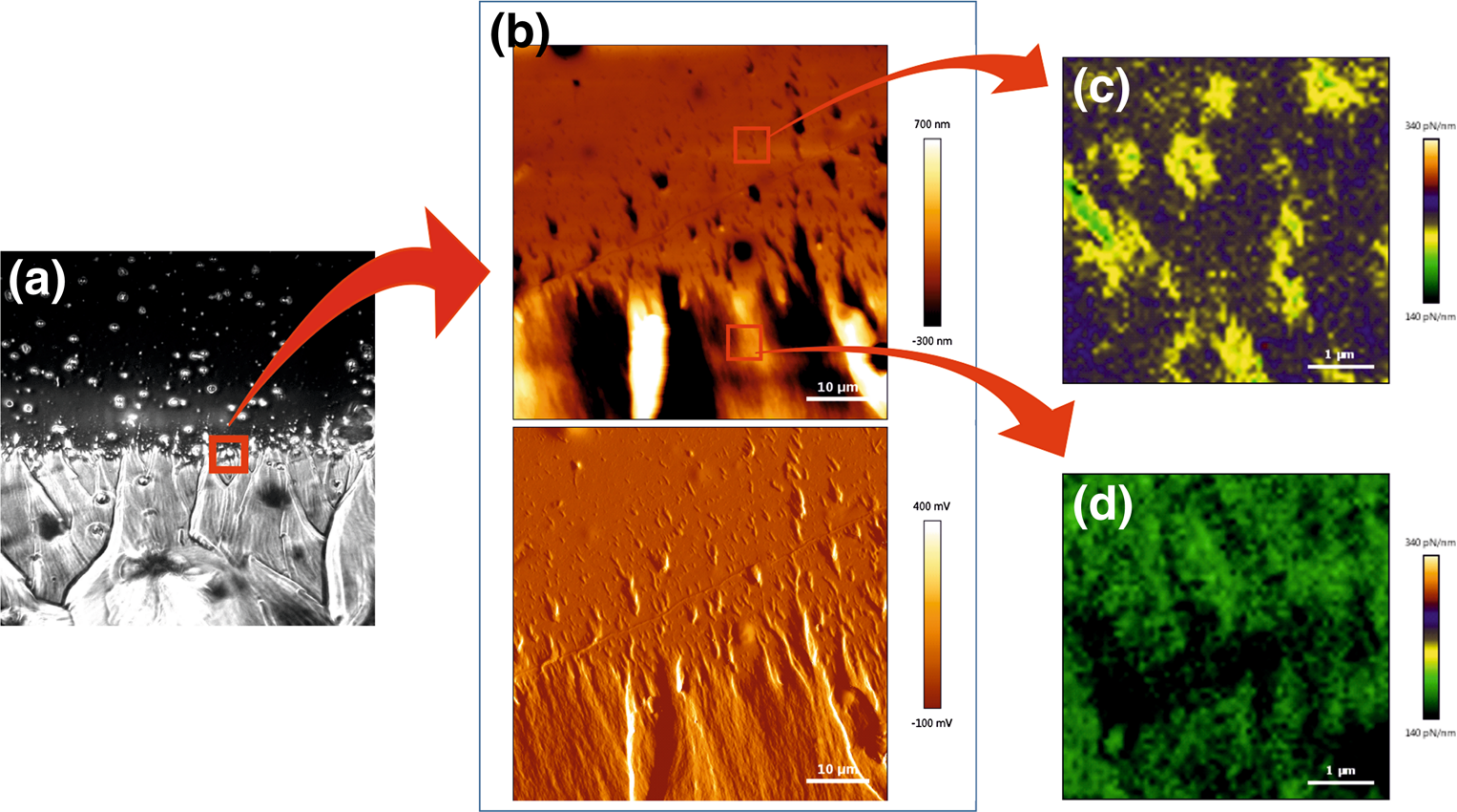
PDMS substrate with discrete elasticity gradient: **a** phase contrast image, **b** AFM topography and error images (scan size: 60 um × 60 um) and elasticity maps on stiffer (**c**) and softer (**d**) region (scan size 5um x 5um)

**Fig. 6:**
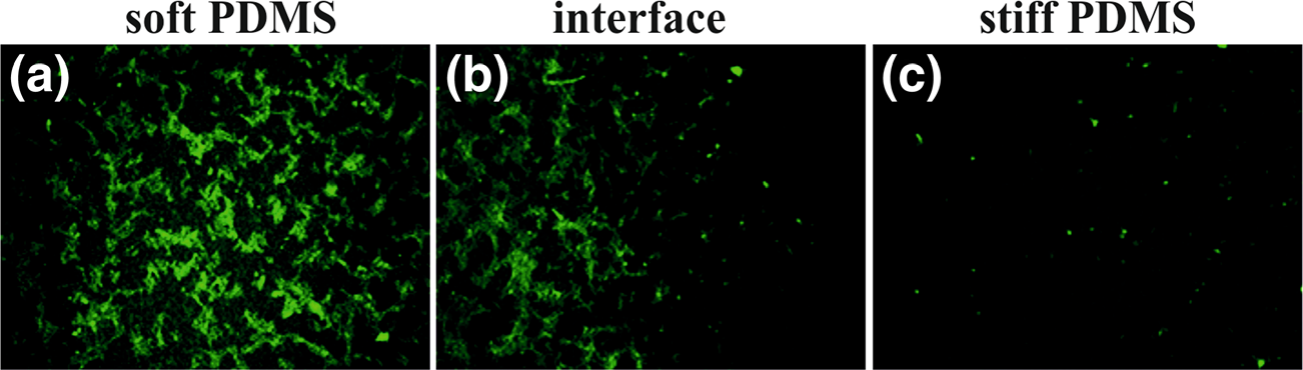
Proliferation of HCV29 cells on substrates with discrete elasticity gradient

**Fig. 7:**
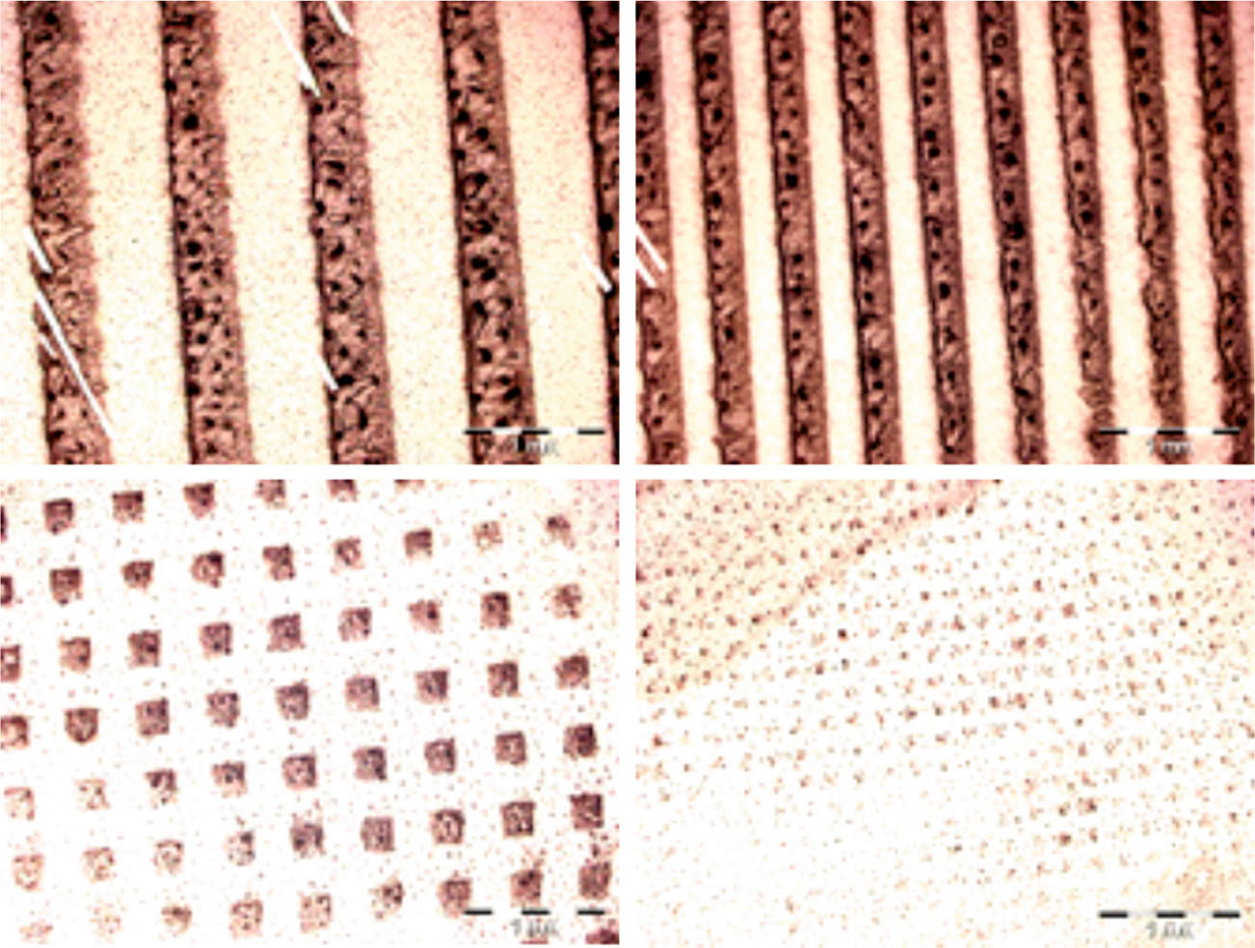
Exemplary PDMS substrates with elasticity pattern of different shapes and dimensions

**Fig. 8:**
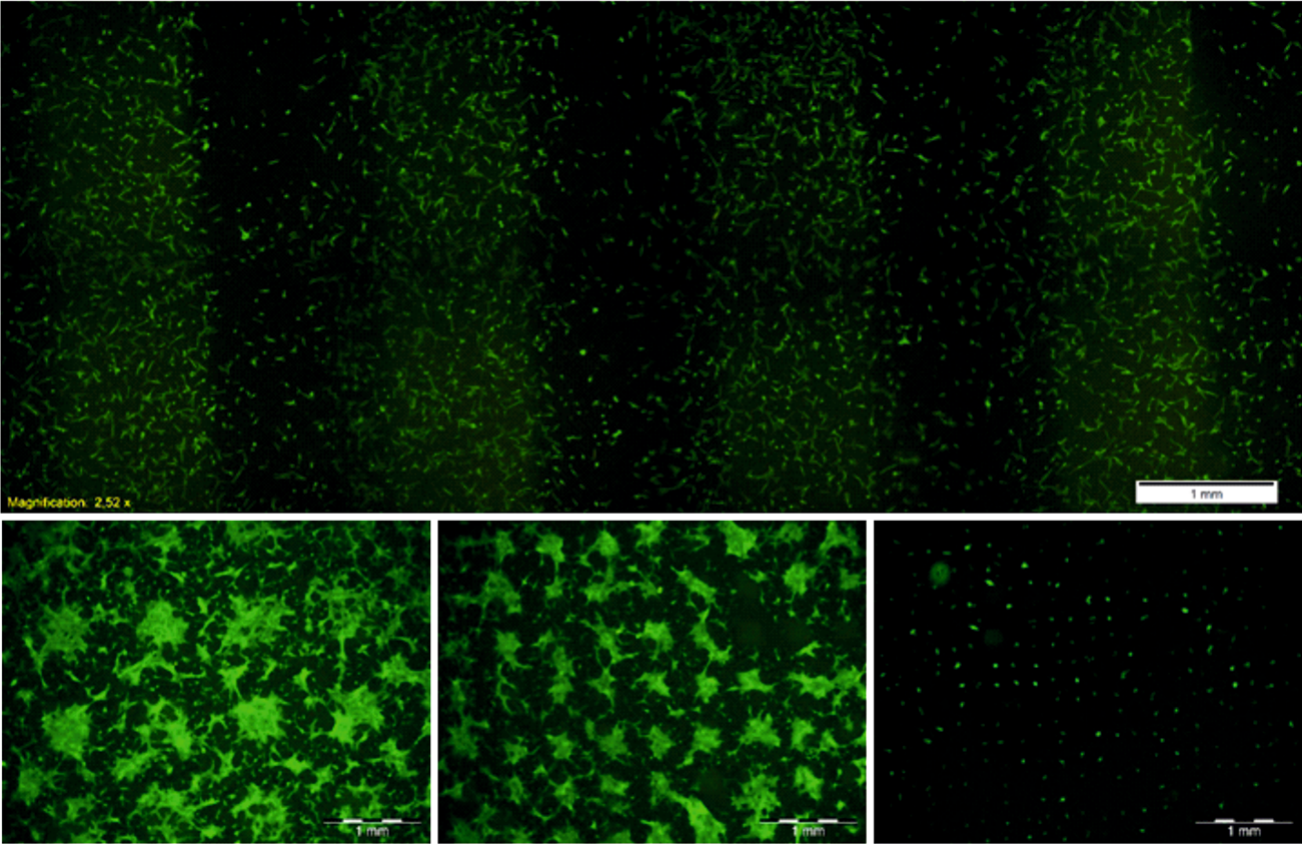
Proliferation of HCV29 cells on PDMS substrates with elasticity pattern of different shapes and dimensions

**Fig. 9:**
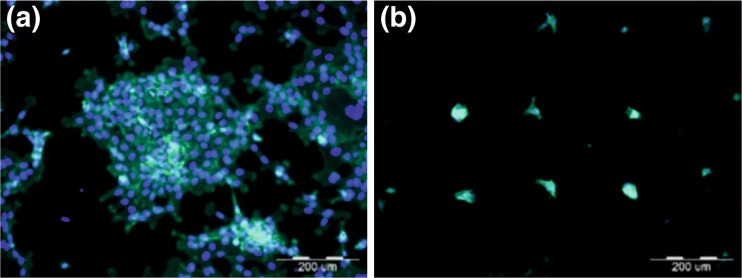
Precise positioning of collective groups (**a**) and individual cells (**b**)

Recorded fluorescence micrographs indicate the elasticity dependent proliferation already after 24 h of incubation. Although for both substrates rare, separated cells can be observed their amount is slightly higher for soft substrate. For longer incubation time (*t* = 144 h), the impact of elasticity becomes significantly more profound and evident. For stiff PDMS the numerous, distinguishable cells are observed, whereas for the soft one the confluent, monocellular layer is formed, with individual cells hardly visible.

To quantify changes in cell growth for PDMS substrates with different elasticity, the number of cells was determined as a function of culture time (Fig. 3). Numerical results validate previous observations. Although for both substrates the number of cells increase monotonically with time of incubation, the proliferation process is approximately twice more effective for cells cultured on soft substrate. After 144 h of incubation number of HCV29 cells increases by 16 times on soft and only 8 times on stiff PDMS.

The results presented here, together with our previous studies on cancerous prostate and melanoma cells (Prauzner-Bechcicki et al. 2015) indicate similarity among cancerous cells. However, it should be noted that effect of stiffness-dependent cytocompatibility is highly cell-specific (Brown et al. 2005; Park et al. 2010).

### Cells on PDMS substrates with continuous elasticity gradient

To analyze more precisely impact of substrate elasticity on proliferation of HCV29 cells, PDMS with continuous elasticity gradient, changing gradually from 0.24 to 1.67 MPa was prepared and used as substrate for cell culture. For each sample, fluorescence micrographs were recorded in three regions – at both ends and in the central part of the sample, i.e. for soft, stiff and ‘medium’ PDMS. However, as the elasticity of the substrate does not change linearly with the UV irradiation time, stiffness of ‘medium’ PDMS is shifted towards lower values (E ~ 0.6 MPa (Raczkowska et al. 2016)). Elasticity maps, determined using quantitative imaging mode and presented in upper row of Fig. 4 show high homogeneity of PDMS mechanical properties over a large scan area.

Representative fluorescence micrographs, recorded after 144h of HCV29 cell culture and presented in bottom row of Fig. 4, show cellular behavior analogous to the one observed on substrates with discrete elasticities - density of cells decreases with increasing stiffness of substrate. For soft and medium PDMS, cells cover whole available surface, creating confluent layer, with single cells hardly distinguishable. In contrast, for stiff substrate the number of cells is significantly reduced and separated individual cells may be still observed.

### Cells on PDMS substrates with discrete elasticity gradient

In living organisms, cells sense strong variations of local elasticity, changing even few orders of magnitude, especially at the interfaces (Engler et al. 2006; Kandow et al. 2007; Seidi et al. 2011; Sunyer et al. 2012; Tse and Engler 2011), which affect strongly their behavior. Therefore proliferation of HCV29 cells was analyzed on substrates with sharp interface between soft and stiff region.

First, to verify formation of sharp elasticity interface, spatial distribution of the PDMS elastic properties was measured in the border area, using QI-AFM mode. In addition to phase contrast image (Fig. 5a), also AFM topography (Fig. 5b) and error (Fig. 5c) micrographs depicted nonlinear but sharp border between two regions of the sample – UV-irradiated and not irradiated. QI-AFM measurements performed in these two areas clearly show difference in the mechanical properties of the substrate. Recorded maps of elasticity depict substrate stiffness significantly higher in upper, not irradiated region of the sample (Fig. 5c), as compared to the bottom one, UV-irradiated for 5 h (Fig. 5d). Obtained results undoubtedly confirm formation of discrete elasticity gradient on PDMS substrates, UV-irradiated through the printed mask. 

Subsequently, these substrates were used to study the influence of discrete elasticity gradient on proliferation process of HCV29 cells. For this purpose large scale fluorescence micrographs were recorded on soft and stiff PDMS region, as well as at their interface (Fig. 6).

In accordance to the previous results, cells proliferate significantly more effective on soft part of the substrate than on the stiff one. The micrographs recorded at the interface of both regions show sharp increase of number of cells, mirroring perfectly the change in elastic properties of the sample.

These results indicate strongly that it should be possible to create substrates with elasticity patterns, enabling precise positioning of cells in desired areas, which is crucial for potential application in cell based biosensors and micro assays.

To test this hypothesis, PDMS substrate with two types of patterns, i.e. alternating soft and stiff stripes and soft squares surrounded by stiff matrix were prepared (Fig. 7) simply by UV-irradiation of PDMS through the printed mask (see Experimental for details).

Then, they were used as substrates to culture HCV29 cells. Fluorescence micrographs, recorded after 72 h of incubation, show preferential proliferation of cells on soft regions of the sample, leading to formation of well-ordered cellular patterns. These results confirm the strong impact of substrate elasticity on cellular behavior, being the driving force enabling precise positioning of analyzed cancerous cells in a wide range of dimensions (Fig. 8, *bottom row*) and over a large area (Fig. 8, *upper row*).

Possibility to create PDMS substrates with elasticity patterns of desired shapes and dimensions enables precise positioning of both, individual cells and groups of them (Fig. 9). This gives the powerful tool to study individual and collective cell – substrate interaction as well as the influence of different external factors on adhesion and proliferation process at the single cell level, which remains the key issue in many biological and medical fields.

## Conclusions

In this work the novel method to create PDMS substrates with continuous and discrete elasticity gradients was introduced. Then, effect of elastic properties on proliferation of bladder cancer cells was analyzed. Obtained results show that studied cancerous cells proliferate significantly more effective on soft PDMS, whereas the stiff one is almost cell-repellant. This strong impact of substrate elasticity on cellular behavior is driving force enabling precise positioning of analyzed cancerous cells.

The simplicity of substrate patterning, based on UV irradiation through printed mask, guarantees enormous variety of accessible patterns for cell positioning over large areas, limited only by printer resolution. Moreover, the stiffness of pattern may be easily modified. Therefore PDMS substrates with elasticity patterns are very promising material for basic studies on effect of varied elasticity on cellular behavior, as well as for potential applications for cell – based biosensors.
